# The Changing Landscape of Voltage-Gated Calcium Channels in Neurovascular Disorders and in Neurodegenerative Diseases

**DOI:** 10.2174/1570159X11311030004

**Published:** 2013-05

**Authors:** Mauro Cataldi

**Affiliations:** Division of Pharmacology, Department of Neuroscience, Reproductive and Odontostomatological Sciences, Federico II University of Naples, Italy

**Keywords:** Neurovascular unit, voltage-gated Ca^2+^ channels, neurodegeneration, beta-amyloid, Parkinson’s disease, Alzheimer’s disease, Multiple Sclerosis.

## Abstract

It is a common belief that voltage-gated calcium channels (VGCC) cannot carry toxic amounts of Ca^2+^ in neurons. Also, some of them as L-type channels are essential for Ca^2+^-dependent regulation of prosurvival gene-programs. However, a wealth of data show a beneficial effect of drugs acting on VGCCs in several neurodegenerative and neurovascular diseases. In the present review, we explore several mechanisms by which the “harmless” VGCCs may become “toxic” for neurons. These mechanisms could explain how, though usually required for neuronal survival, VGCCs may take part in neurodegeneration. We will present evidence showing that VGCCs can carry toxic Ca^2+^ when: a) their density or activity increases because of aging, chronic hypoxia or exposure to β-amyloid peptides or b) Ca^2+^-dependent action potentials carry high Ca^2+^ loads in pacemaker neurons. Besides, we will examine conditions in which VGCCs promote neuronal cell death without carrying excess Ca^2+^. This can happen, for instance, when they carry metal ions into the neuronal cytoplasm or when a pathological decrease in their activity weakens Ca^2+^-dependent prosurvival gene programs. Finally, we will explore the role of VGCCs in the control of nonneuronal cells that take part to neurodegeneration like those of the neurovascular unit or of microglia.

## INTRODUCTION

A strong increase in [Ca^2+^]_i_ may cause the saturation of mitochondrial buffering capacity, mitochondrial damage and, eventually, neuronal death [1, 2]. This is considered the main mechanism responsible for neuronal cell death in neurodegeneration. The first evidence that Ca^2+^ overload is a death-triggering signal came from experiments performed in nonneuronal cells like hepatocytes and skeletal muscle cells [3, 4]. In 1984, Roger Simon discovered that a massive Ca^2+^ build-up takes place in the hippocampus after an ischemic insult. This finding suggested that Ca^2+^ overload could be lethal also in neurons and marked the beginning of the *Ca^2+^ theory* *of neurodegeneration* [5]. Up to date, hundreds of papers have been published on this issue. Since the early days, it was proposed that the pharmacological blockade [Ca^2+^]_i _overload could rescue neurons from death. Therefore, there was (and there still is) a formidable interest in identifying excess Ca^2+^ sources in neurodegenerative diseases. At the time, the most obvious candidates were the varied families of ionotropic glutamate receptors and voltage-gated Ca^2+^ channels (VGCCs) (see Table **1** and references [6] and [7] for detailed reviews on these channels). They were, indeed, the best characterized Ca^2+^ influx pathways in neurons. Many papers showed benefits of a new class of drugs blocking L-type Ca^2+^ channels, the Ca^2+^ channel blockers (CCBs), in diverse preclinical models of neurodegenerative disorders. Soon, however, serious doubts arose about the importance of VGCCs in neurodegenerative cell death and other ion channels and transporters appeared way more important in this process. Among them, glutamate receptors were initially thought the most important. More recently, fancier channels and transporters like acid sensitive ion channels (ASICs), transient receptor potential cation channel, subfamily M, member 7 (TRPM7), or sodium calcium exchanger (NCX) [8, 9] emerged as major death effectors in neurons. The failure of some clinical trials with CCBs in neurodegenerative diseases like stroke [10] was an important reason to abandon the idea that VGCCs are relevant in neurodegeneration. Moreover, several studies *in vitro* suggested that these ion channels were not the right channels to carry a “toxic” Ca^2+^ influx into neurons. However, the old idea that VGCCs could be targeted to treat neurodegenerative and neurovascular diseases was never abandoned. Recent data provide new evidence in its support [11]. In the present review, we will go through these developments, and we will show how they are changing the old dogma that VGCCs simply act as a general and “nonspecific” source of excess Ca^2+^ in neurons. To be specific, we will review evidence suggesting that neuronal VGCCs activation, though usually ineffective in killing neurons, may become lethal in specific neuronal subpopulations or in specific disease states. We will also examine how these channels may become lethal for neurons even without carrying excess Ca^2+^. Finally, we will describe the role of VGCCs on nonneuronal cells that take part to neurodegeneration, like vascular smooth muscle cells, microglia or macrophages. 

### 1 Conditions that Confer to VGCCs the Ability to Carry Toxic Ca^2+^ Loads

**Fig. (1):**
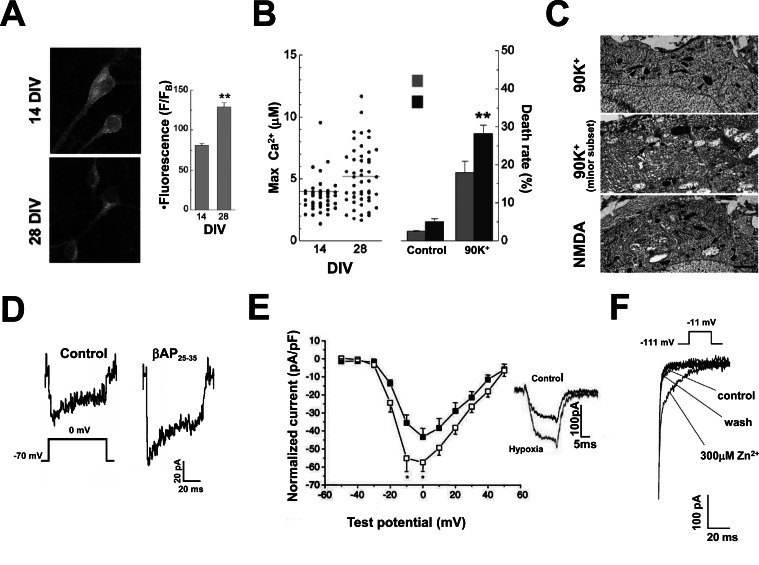
**Factors causing a pathological VGCC current increase in neurodegenerative diseases.** *A, B and C*, neurons aged *in vitro* carry larger VGCC currents than young neurons and are more vulnerable to death. *A*, confocal fluorescence images of hippocampal neurons incubated with anti Ca_V_1.2 primary antibodies and FITC-conjugated secondary antibodies. Note that Ca_V_1.2 immunoreactivity is higher in 28 than in 14 DIV cultured neurons. The bar graph in the inset reports the mean±SEM of the fluorescence intensities measured in 28 DIV (n=24) and 14 DIV (n=46) neurons. *B*, the scatter plot on the left reports the values of maximal [Ca^2+^]_i_ increase elicited by a 90 mM K^+^- containing extracellular solution in single 14 and 28 DIV neurons. The bar graph on the right shows the fractional cell death in the same conditions. Note that cell death is higher in 28 DIV than in 14 DIV neurons. *C*, electron micrographs obtained in 28 DIV hippocampal neurons freeze-dried and sectioned immediately after being exposed to either 90 mM K^+^ or 100 µM NMDA. Note the presence of mitochondrial swelling (arrows) both in NMDA and in 90 mM K^+^ treated cultures. Arrowheads indicate normal mitochondria for comparison (*A*, *B* and *C* reproduced with permission from Stanika *et al.*, 2012 [21]). *D*, βAPs increase VGCC currents in pyramidal hippocampal neurons. Cultured hippocampal neurons were exposed for 16 hours to 10 µM βAP_25-35_ before patch-clamp recording (reproduced with permission from Ueda *et al.*, 1997 [44]). *E*, chronic hypoxia causes an increase in VGCC current amplitude. The graph shows voltage to current plots obtained in rat cerebellar neurons cultured under normoxic (■) and hypoxic conditions (□). Chronic hypoxia was obtained by incubating the neurons for 24 h before patching in a 2.5% O_2_, 5%CO_2_ and 92.5% N_2_ atmosphere. The holding potential was set at -90 mV and currents were elicited by voltage steps up to 0 mV. The inset shows superimposed the means of all the traces recorded in control and hypoxic neurons as indicated (reproduced with permission from Webster *et al.*, 2006 [52]). *F*, Zn^2+^ slows down current deactivation kinetics in recombinant Ca_V_3.3 T-type channels. The panel shows the tail currents recorded upon membrane repolarization in control condition, in the presence of 300µM Zn^2+^ and after washing out this transition metal in a Ca_V_3.3 stably expressing HEK-293 cell held at -111mV and stepped up to -11mV (voltages corrected for junction potential). Note that in the presence of Zn^2+^ current deactivation is greatly slowed down (reproduced with permission from Cataldi *et al.*, 2007 [87]).

As originally intended, the *Ca^2+^ neurotoxicity*, or *Ca^2+^ load*, theory assumed that neurons die whenever their [Ca^2+^]_i_ becomes higher than a critical threshold [12]. According to this theory, every Ca^2+^ source, also including VGCCs, may induce cell death if it carries large Ca^2+^ loads into neurons. The important experimental work of Michael Tymianski and his group seriously questioned this idea and led to the influential *Ca^2+^ source specificity* theory [13]. Briefly, this theory assumes that Ca^2+^ is toxic for neurons if it may activate specific death triggering systems. This may happen when Ca^2+^ enters the neurons through specific pathways that are close to these death effectors. According to the *Ca^2+^ source specificity* theory and in contrast with the *Ca^2+^ load* theory, not all the Ca^2+^ sources are capable of triggering cell death. Importantly, evidence suggested that VGCCs are not the right channels to kill neurons. Specifically, only 20% of cultured spinal [13] and cortical [14] neurons died when these channels were activated with 50 mM K^+^ given alone or with the L-type channel activator Bay-K 8644. On the contrary, NMDA agonists killed neurons in a dose-dependent manner. Cell death was also proportional to the increase in [Ca^2+^]_i_ caused by these compounds [14]. These data suggested that whereas NMDA channels may trigger neuronal cell death VGCCs may not. Interestingly, NMDA agonists caused more cell death than high K^+^ even in cultures that showed a similar increase in [Ca^2+^]_i_ in response to these treatments [14]. This suggested that the Ca^2+^ carried by VGCCs is not the “right” Ca^2+^ to kill neurons. It was suggested, indeed, that Ca^2+^ entering through these channels has different physiological roles than triggering cell death. Specifically, it activates gene programs, like that involving CREB, that promote cell survival [15-18]. Several explanations have been proposed to explain why, differently from VGCCs, NMDA receptors have a privileged role in neuronal death. The most popular of these hypotheses is that they are close to neuronal nitric oxide synthase (nNOS) in the postsynaptic density [19, 20]. So, Ca^2+^ entering through NMDA receptors activates nNOS leading to the production of toxic peroxynitrites [20]. These toxic compounds could further increase Ca^2+^ overload by activating TRPM7 channels [20]. In conclusion, the *source specificity theory* assumes that VGCCs cannot cause death in neurons because they do not couple with death effectors like nNOS. Recent data questioned these conclusions. They showed, indeed, great differences in the [Ca^2+^]_i_ responses and in the cell damage caused by depolarization in different single cortical and hippocampal neurons [21] (Fig. **1A**-**D**). In most of the neurons, membrane depolarization with high K^+^ caused an [Ca^2+^]_i_ response lower than after NMDA receptor stimulation and no cell damage [21]. On the contrary, in a small group of neurons, it caused a steady increase in [Ca^2+^]_i_ up to values close to those induced by NMDA agonists and signs of impending cell death [21]. These included a massive build-up of Ca^2+^ ions into the mitochondria, mitochondrial depolarization, and relevant structural changes in these organelles [21] (Fig. **1C**). Thus, it appears likely that in a subset of neurons, membrane depolarization may cause a marked increase in [Ca^2+^]_i_ and cell death. In keeping with this hypothesis, neuronal aging *in vitro* causes a parallel increase in the percentages of dying neurons, of neurons showing high L-type VGCC density and of neurons showing high [Ca^2+^]_i_ responses to depolarization (Fig. **1A** and **B**). Based on these data, Stanika *et al*. [21] proposed that VGCCs do not cause neuronal death because in the majority of neurons they do not carry enough Ca^2+^. Indeed, in cells where their activation causes large Ca^2+^ loads, membrane depolarization triggers a mitochondrial-dependent cell death [21]. These data suggest that VGCCs could promote neurodegeneration if their density or activity is increased over the average. This raises the question of whether this phenomenon happens in specific neurodegenerative diseases. VGCC channelopathies are a good answer to this question. Indeed, in these diseases, mutations in VGCC genes cause changes in the activity of these channels and death of selected neuronal populations. These rare disorders are a paradigmatic example of the involvement of VGCCs in neurodegeneration. However, we will not discuss about them because they have been the object of excellent reviews where the interested reader can find an in-depth analysis of their pathophysiology [22-24]. Instead, we will focus, on common neurodegenerative disorders not caused by mutations in VGCC genes, like Alzheimer’s disease (AD) or Parkinson’s disease (PD). We will distinguish two generally different situations leading to VGCC-dependent cell death in these diseases. First, we will examine conditions where specific pathological stimuli increase the expression or activity of VGCCs, thus killing neurons otherwise refractory to depolarization-induced death. Second, we will explore conditions in which depolarization-induced cell death occurs in neurons that are intrinsically more susceptible to VGCC-dependent cell death. In these cases, factors increasing VGCC activity or density are not required to induce cell death. Specifically, this happens in neurons relying on these channels for action potentials generation or propagation. 

#### 1a Conditions Leading to a Pathological Increase in the Expression or Activity of VGCCs

Aging increases the risk of Alzheimer’s disease, and vascular dementia [25-27]. These diseases often coexist in the same patient giving rise to a mixed Alzheimer’s disease and vascular dementia syndrome [28]. Furthermore, neurovascular disorders are an important risk factor for different forms of dementia [29-31]. So, the incidence of this disease is doubled in subjects aged less than 85 years that previously suffered from brain ischemic episodes [29-31]. Several findings suggest that a reason for linking these conditions the one to the other and all to aging could be a pathological increase in L-type VGCC currents. 

The idea that normal **aging **does increase the activity of L-type Ca^2+^ channels was proposed more than 20 years ago. It is supported by a wealth of experimental data. For instance, in aged animals, neuronal whole-cell L-type currents are larger than in younger animals [32]. Besides, Ca^2+^-dependent action potentials or Ca^2+^-dependent after-hyperpolarization, that depend both on these currents, increase as well [32]. Importantly, the increase in L-type currents caused by aging also leads to a potentiation of Ca^2+^-dependent synaptic depression [34]. This could have a role in cognitive impairment of the elderly [33]. Cultured aged neurons are more vulnerable to death than younger neurons and their higher vulnerability is at least in part L-type channel dependent [34]. In keeping with this evidence, a maximal depolarizing challenge evokes [Ca^2+^]_i_ responses larger than 5 µM in a higher percentage of 28 DIV “aged” than of 14 DIV “young” neurons [21]. This finding suggests that L-type dependent Ca^2+^ influx could be responsible for the high vulnerability of aged neurons [21]. Our knowledge of the mechanism responsible for the increase in L-type currents in aged neurons is still limited. We know that this process does not depend on changes in L-type channel expression. Indeed, the expression of the major L-type isoform, Ca_V_1.2, strongly decreases in aging whereas the less represented isoform Ca_V_1.3 only marginally increases [35-36]. Instead, the available evidence points to an increase in L-type channel activity whose origin remains, however, obscure. Davare and Hell reported evidence that an increase in protein kinase A (PKA)-mediated channel phosphorylation could be involved [37]. According to recent evidence, the imunophilin FKBP1b could also have a role in aging-induced L-type channel potentiation [38]. The levels of this protein, that binds to ryandodine receptor type 2 in the brain and keeps it closed [39, 40], decrease in normal aging [41]. Aging-induced changes in FKBP12.6 were reproduced by RNA interference both *in vitro*, in cultured neurons, and *in vivo*, in young rats [38]. Intriguingly, in these conditions, both L-type currents and Ca^2+^-dependent slow after-hyperpolarization greatly increased [38].

**β-Amyloid Peptides (βAPs)** accumulate in Alzheimer’s disease resulting in amyloid plaques. These peptides are considered responsible for neuronal cell death in this disease. Importantly, βAPs markedly increase VGCC currents. This was first demonstrated in differentiated mouse N1E-115 neuroblastoma cells [42]. Later, this effect of βAPs was observed also in hippocampal and cortical neurons and in cerebellar granules [43-45] (Fig. **1D**). Early studies reported a significant potentiation not only of L-, but also of N- and P-type currents upon βAP exposure [46]. More recently, data have been reported suggesting that βAPs do inhibit P-type currents and that this effect could be relevant in causing the cognitive disorders typical of Alzheimer’s disease [47]. βAP also inhibits recombinant P/Q type channels expressed in Xenopus oocytes [48]. The reason of the discrepancy among these studies is presently unknown. Aggregation status influences the ability of βAP to modify VGCC currents. The “toxic” forms of these peptides, the βAP oligomers, activate VGCCs much more than the aggregated forms that are not toxic [46]. The mechanism by which βAPs increase VGCC currents remains elusive. However, L-type channel activation could depend on βAP-induced free radical generation [44] or channel phosphorylation by MAP-kinases [49]. Recent work on recombinant L-type channels showed that βAP_25-35_ affects channel trafficking to the plasma membrane by binding to the accessory β3 subunits [50]. 

L-type currents increase also in the presence of** chronic hypoxia** that occurs, for instance, in conditions like atherosclerotic dementia. The first evidence of the increase in L-type currents was obtained in PC12 cells [51] and in cerebellar neurons [52] cultured in a low-oxygen atmosphere (Fig. **1E**). Some evidence suggests that βAPs are responsible for the increase in L-type channel activity caused by hypoxia. This could explain why AD and cerebrovascular disorders often occur together in the same patients. Brain ischemia increases the expression of amyloid precursor protein (APP) mRNAs and proteins [53, 54]. Specifically, mRNAs for APP isoforms with a Kunitz-type serine protease inhibitor domain (KPI) build-up in ischemia [53]. Also the activity of β- and γ-secretase is enhanced in this condition [55]. The following evidence supports the hypothesis that βAP is responsible for the increase in L-type channel activity caused by hypoxia. First, the pharmacological blockade of β- or γ-secretase [56], or the immunoneutralization of βAP with monoclonal antibodies prevent the effect of hypoxia on VGCCs [51]. Second, the exposure to βAP_25-35_, βAP_1-40_ or βAP_1-42_ replicate this effect [51, 52]. L-type channels carry most of the inward currents induced by hypoxia. On the contrary, the contribution of other HVA subtypes like N-type channels is negligible [51, 52]. Nonspecific ion channels made by βAPs dissolved into the plasma-membrane carry only a minor, Cd^2+^-resistant, component of hypoxia-induced currents [57]. Hypoxia does not induce changes in the gating properties of L-type channels. Western blot and immunocytochemistry data show that, instead, it increases channel density in the plasma-membrane [56]. This effect depends on an enhanced translocation of L-type channels to the plasma membrane [56]. It is prevented, indeed, by drugs that disrupt vesicle recycling [56]. Antioxidant drugs prevent the effect of hypoxia on L-type channels [58]. Furthermore, hypoxia does not affect L-type currents in cell lines that have been depleted of mitochondria with ethidium bromide [58]. These findings suggest that radical oxygen species (ROS) have a role in L-type current potentiation by hypoxia. Importantly, the levels of these compounds are high in hypoxia because mitochondrial respiration is impaired [58]. The increase in L-type channel activity induced by βAP_1-40_ is not affected when ROS levels are decreased. These data suggest that hypoxia promotes, by a ROS-dependent mechanism, the generation of βAPs that, then, modulate L-type channels [56]. Recent evidence shows that, indeed, hypoxia increases the transcription of β-secretase 1 (BACE1) *via *ROS and the oxygen-sensitive transcription factor hypoxia-inducible factor 1 (HIF-1) [59]. Coimmunoprecipitation experiments suggest that βAPs could affect L-type channels binding to the channel proteins [56]. In keeping with this hypothesis, in heterologous expression systems βAPs affect L-type channel trafficking by interacting with their β3 subunits [50]. 

Do all these data support the use of CCBs in Alzheimer’s disease or in dementia syndromes? The hypothesis that CCBs could be helpful in these conditions was proposed already in the early days of the Ca^2+^ neurotoxicity theory. At the time several studies showed the protective effects of CCBs on βAP-induced cell death in neurons cultured *in vitro* [60, 61]. Other studies, however, did not confirm these findings [62]. Several epidemiological studies evaluated whether patients treated with CCBs for cardiovascular disorders are protected from AD and dementia. They yielded, however, contradictory results. Some studies but not others showed, indeed, that CCBs improve AD symptoms in the short term and prevent or delay, disease progression [63]. These inconsistencies could be due to differences among the different CCBs. For instance, some CCBs but not others efficiently cross the blood-brain barrier. Also, some CCBs may affect βAP toxicity by mechanisms unrelated to VGCC blockade. For instance, nimodipine promotes βAP_1-42_ secretion in a L-type channel-independent way [64]. Also, bepridil affects βAP processing by interfering with endosome pH [65]. Finally, some CCBs like verapamil reduce the clearance of βAP because they block P-glycoprotein, the pump responsible for its removal from the brain [66-68]. Nivaldipine [63, 69] and isradipine [70] are the most promising CCBs for the treatment of AD and other forms of dementia. Specifically, nivaldipine retarded the progression of cognitive decline in patients with mild cognitive impairment [71, 72]. Besides, it stabilized cognition and improved executive function in AD patients. This effect was higher in patients not showing the APOE ε4 phenotype [69]. Isradipine is neuroprotective for MC65 cells [73, 74]. This cell line represents a model that reproduces *in vitro* βAP neurotoxicity more faithfully than cultured neuronal cells exposed to exogenous βAP [73, 74]. It is a neuroblastoma cell line stably transfected with amyloid precursor protein (APP)-C99 under control of a tetracycline (Tet)-repressor cassette. Therefore, it releases βAPs and undergoes βAP-dependent cell death when tet is removed from the culture medium [73]. Isradipine prevented βAP neurotoxicity also in several AD models *in vivo* including drosophila, the moth Manduca sexta and 3xTgAD mice, which harbor the presenilin-1 (PS1) (M146V), APPswe and tau (P301L) transgenes [74]. Despite this encouraging evidence, the blood pressure lowering effect of CCBs may be of concern for it lowers brain perfusion. We will discuss further the neurovascular effects of CCBs in AD in section 3a. A more rational approach than blocking L-type channels would be to correct the molecular mechanisms responsible for the increase in their activity or expression. Unfortunately, although some information on this issue begins to be available, no drug therapy specifically directed against these targets has been developed yet.

Membrane potential becomes depolarized in neurons after hypoxia *in vitro* or **ischemic stroke** *in vivo*. Therefore, VGCCs are expected to be strongly activated in these conditions (see section 3a for further details) [75]. This suggested that VGCC blocking drugs could be neuroprotective in stroke. It was also reported that these compounds may counteract Ca^2+^-dependent hypoxic vasoconstriction and improve the perfusion of ischemic brain [76]. A wealth of preclinical data confirmed the neuroprotective properties of L-, N- and P-type channels blockers [77]. However, CCBs failed in several clinical trials on stroke in humans [10]. Nowadays, it is clear that they are not effective in this disease. On the contrary, the information available is still insufficient to establish the role of N- or P-type channel blockade in brain ischemia [77, 78]. A likely reason of the failure of L-type channel blockers in stroke is that, in the *ischemic core*, Ca^2+^ enters the neurons through many other ion channels and transporters [8,9]. In this part of the brain, cerebral blood flow (CBF) dramatically decreases after stroke and neurons die almost immediately after the ischemic insult [8, 9]. Around the ischemic core there is a region, the *penumbra*, where CBF, though lower than normal, still preserves ionic homeostasis. Neurons in the penumbra die, indeed, only hours after the initial ischemic event. Therefore, there is enough time to rescue neurons this region of through pharmacological interventions. Surprisingly, L-type currents in the neurons of the penumbra are not increased, as expected, but significantly decreased respect to control conditions [79]. The decrease in L-type currents occurs in CA1 hippocampal neurons, that are sensitive to ischemia, but not in CA3 neurons, that are more resistant to this insult [79]. Protein expression of L-type channels is comparable in the ischemic penumbra and in control tissue. On the contrary, L-type single channel open probability is significantly decreased [79]. This effect could depend on the oxidation of the channel [79], a common mechanism affecting ion channel activity in stroke [80]. What is the pathophysiological meaning of the decrease in L-type channel currents in the penumbra? The authors of the cited paper speculated that it contributes to delayed neuronal cell death [79]. A decrease in Ca^2+^ influx through L-type channels could, indeed, blunt neuroprotective gene programs like that mediated by CREB [15-18]. Consistent, the L-type channel agonist Bay-K 8644 protects neurons in models *in vitro* and *in vivo* of brain ischemia [79]. Recently, the group of Ricardo Dolmetsch in Stanford reported that glutamate exposure *in vitro* causes a decrease in the density of L-type channels in the plasma membrane [80]. This decrease occurred because of the internalization and the lysosomal degradation of the Ca_V_1.2 pore-forming subunits [80]. This process involves the following consecutive steps. First, Ca_V_1.2 binds to Pikfyve, an enzyme that generates phosphatidylinositol (3,5)-bisphosphate (PtdIns(3,5)P2). Second, Ca_V_1.2-containing endosomes become enriched with PtdIns(3,5)P2. Finally, PtdIns(3,5)P2-enriched vesicles are targeted to lysosomes [80]. Because they are degraded in lysosomes, Ca_V_1.2 containing channels cannot be recycled to the plasma-membrane. This causes a persistent decrease in L-type currents. Therefore, glutamate targets Ca_V_1.2 channels to lysosomal degradation. On the contrary, after depolarization, Ca_V_1.2 channels are internalized but not targeted to lysosomes. Therefore, they may rapidly return to plasma membrane [81]. Differently from what reported by Li [79], glutamate-induced Ca_V_1.2 degradation seems to protect neurons from Ca^2+^ overload and cell death. Pikfyve silencing *in* *vitro* causes, indeed, an increase in glutamate-induced cell death presumably because more Ca^2+^ can enter the neuron through L-type channels [80]. In keeping with this hypothesis, old data show, indeed, that L-type channels become activated after NMDA receptor stimulation and contribute to glutamate-induced Ca^2+^ overload and cell death [14]. Also, glutamate-induced cell death in Pikfyve silenced neurons can be prevented by L-type channel blockade with nimodipine [80]. Pikfyve-dependent decrease in L-type channel density could be a key neuroprotective mechanism in glutamate-dependent forms of neurodegeneration such as in stroke or in cell death after seizures. This intriguing hypothesis remains, however, to be directly explored *in vivo*. 

T-type or low voltage-activated (LVA) channels are another class of VGCC that could have a role in **ischemic neuronal cell death**. These channels, differ from HVA Ca^2+^ channels under many respects including permeation and gating properties [82, 83]. Importantly, in LVA but not in HVA channels, the voltage dependence of activation and the voltage dependence of inactivation overlap at voltages around -50 mV [82]. This implies that when membrane resting potential assumes values inside this window of overlap, a definite fraction of T-type channels stays stably open and can carry excess Ca^2+^ inside the neuron. This could happen in the early phases of ischemia when neurons start to depolarize and their resting membrane potential move into the T-type channel window [84]. Therefore, it has been suggested that T-type channels could be an important Ca^2+^ influx path in ischemic neurons. The only evidence that T-type channels take part to brain ischemia comes from experiments performed with the unselective T-type channel blockers mibefradil, pimozide and nickel [84, 85]. These drugs decreased ischemic neuronal cell death in brain slices undergoing oxygen-glucose deprivation [85]. Also, they lessened cell loss in the hippocampus of rats subjected to global ischemia [86]. We still ignore which of the three T-type channel isoforms, Ca_V_3.1, Ca_V_3.2 and Ca_V_3.3 [82], takes part to ischemic death in neurons. Further experiments in knock-out animals or *in vitro* silencing studies will be necessary to clarify this point. 

Transition metals cause an increase in the activity of T-type channels that could be relevant in brain ischemia. In particular, we showed that Zn^2+ ^affects the gating properties of the Ca_V_3.3 isoform of T-type channels [87]. To be specific, it markedly slows down their deactivation [87]. This is expected to increase the amount of Ca^2+^ entering the neurons on membrane repolarization. The importance of this mechanism in neurodegeneration remains to be proved. However, it is worth to remind that Zn^2+^ ions have a well established role in neurodegeneration. These ions, indeed, are coreleased with glutamate and cooperate with this neurotransmitter in causing neuronal cell death in conditions like brain ischemia and AD [88, 89]. 

#### 1b VGCC “Toxicity” in Neurons Showing VGCC-Dependent Action Potentials

**Fig. (2):**
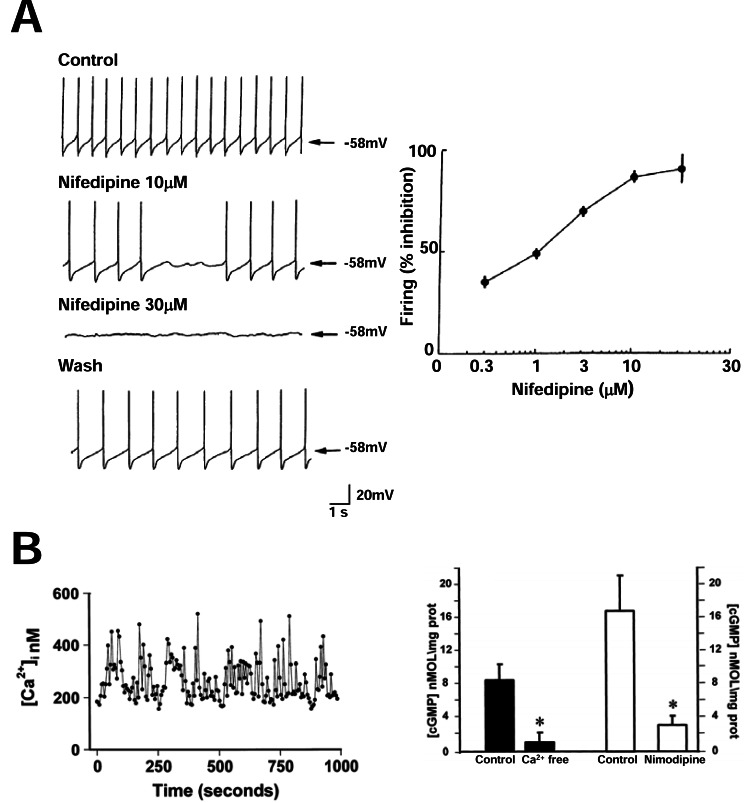
**High Ca^2+^ loads in cells showing Ca^2+^-dependent oscillations.** *A*, SN_C_ neurons fire spontaneous Ca^2+^-dependent action potentials. The panel shows representative current clamp recordings obtained in a SN_C_ neuron recorded from an acute rat brain slice in basal condition, in the presence of two different concentrations of nifedipine and after washing out this CCB. Note that in the presence of nifedipine spontaneous action potential generation is prevented. The plot on the right of the panel shows the dose-dependence of the suppression of SN_C_ neuron firing by nifedipine (reproduced with permission from Mercuri *et al.*, 1994 [99]). *B*, Ca^2+^-dependent oscillations in pituitary GH3 cells are coupled with the activation of the NO-cGMP cascade. The trace on the left of the panel shows typical [Ca^2+^]i oscillations in a single fura2-loaded GH_3_ cell. The bar graph on the right shows the effect on intracellular cGMP concentrations of the removal of extracellular Ca^2+^ or of the incubation with the CCB nimodipine, two conditions that abrogate [Ca^2+^]_i_ oscillations in GH3 cells; *= p<0.05 (reproduced from Cataldi *et al.*, 1998 [96] with permission).

In the previous sections we showed that specific factors may cause neuronal cell death by increasing VGCC density or activity. Now we will review evidence that selected neuronal populations are intrinsically susceptible to Ca^2+^ overload. This may happen in neurons that show a ***Ca^2+^-dependent pacemaker activity***. These cells undergo large oscillations in [Ca^2+^]_i_. Therefore, even in physiological conditions, they have average [Ca^2+^]_i_ significantly higher than other neuronal populations. Pacemaking is common in neurons but it is Ca^2+^-dependent only in a minority of cases. Indeed, in most CNS neurons I_h_ channels or TTX-sensitive persistent Na^+^ currents are responsible for pacemaking [90]. In few neuronal populations [90] repetitive action potential generation relies on VGCCs. This resembles the pacemaking of nonneuronal cells like sinoatrial cardiac cells [91] and pituitary cells [92, 93]. Ca_V_3 low-voltage activated T-type channels or Ca_V_1.3- containing L-type channels are the VGCCs involved in this process [82, 94]. These channels open at subthreshold voltages and, therefore, may trigger action potentials [82, 95]. Thalamic neurons are typical examples of T-type dependent pacemaker cells [82]. Instead, Ca_V_1.3 channels are responsible for spontaneous firing in substantia nigra pars compacta (SNc), in midspiny striatal neurons, in adrenal chromaffin cells and in cochlear immature inner hair cells [94] (Fig. **2A**). In pacemaker cells, high Ca^2+^ amounts enter the cytoplasm at each spike. Therefore, these neurons are exposed to higher Ca^2+^ loads than non-oscillating cells or cells with nonVGCC-dependent pacemaking. Also, in Ca^2+^-dependent pacemaker cells, Ca^2+^ influx through L-type channels causes NO generation and cGMP increase [96] (Fig. **2B**). These data suggest that neurons with Ca^2+^-dependent pacemaker cells could be especially susceptible to cell death. The group of D.J. Surmeier showed that this occurs in SNc neurons [97]. SNc neurons have a special relevance in neurodegeneration because they are the first to degenerate in PD. SNc neurons fire spontaneous Ca^2+^-dependent action potentials at a frequency of 2-4 Hz [98]. Classical studies showed that in these neurons action potential generation depends on L-type channel opening. Dihydropyridines like nifedipine, nimodipine or isradipine make, indeed, it stop [97-100] (Fig. **2A**). 

Elegant studies showed that Ca^2+^-dependent pacemaking is responsible for the high susceptibility of SNc neurons to neurodegeneration. Briefly, Chan and coworkers were interested in establish whether Ca_V_1.3 channels are responsible for pacemaking in SNc neurons that strongly express these channels [97]. Therefore, they studied the spontaneous electrical activity of SNc neurons from Ca_V_1.3 knock-out mice [97]. To their surprise they found that these cells were still discharging at the expected frequency [97]. However, differently from controls, in knock-out mice pacemaking depended on Na^+^ channels and I_h_ and not on L-type channels [97]. This form of pacemaking is typical of juvenile SNc neurons. This suggested that the developmental switch from Na^+^- to Ca^2+^-dependent pacemaking does not take place in Ca_V_1.3 knock-out mice [97]. SNc neurons from Ca_V_1.3 knock-out mice are more resistant to to neurotoxic compounds, like the parkinsonigen agent rotenone [101], than those from their littermates [97]. This suggested that Ca^2+^-dependent pacemaking has a role in SNc susceptibility to death. Consistent with this idea, isradipine, an L-type channel blocker with some selectivity for Ca_V_1.3 channels [102], protect SNc neurons from parkinsonigen agents. Interestingly, isradipine forces SNc neurons from wild type mice to switch to a Na^+^-dependent juvenile pacemaking pattern [97]. Isradipine is also neuroprotective in models *in vivo* of the disease based on rotenone, MTPP or 6-OH dopamine toxicity [97-103]. Isradipine also prevents abnormal movements in the 1-methyl-4-phenyl-1,2,3,6-tetrahydropyridine (MPTP) model of PD diskinesia [104]. These findings suggest that CCBs could be helpful in PD. However, the available clinical evidence do not support this hypothesis [105]. For instance, a large retrospective analysis of Ontario’s health care databases did not show any evidence that long-term treatment with dihydropyridines for hypertension could lower the risk of PD [106]. This could be due to the fact that most of currently marketed CCBs only weakly block Ca_V_1.3 channels [102]. The only exception could be isradipine [102] that, therefore, is currently evaluated for PD. The results of a pilot dose escalation study using this drug in early PD have been published already [107]. A randomized double blinded phase II study, the “Safety, Tolerability and Efficacy Assessment of Dynacirc CR in Parkinson’s Disease (STEADY-PD)” (http://www.clinicaltrials.gov/ct2/show/NCT00909545?term=STEADY-PD&rank=1) is underway and its results are eagerly awaited. 

The evidence reviewed above suggested that aging SNc neurons degenerate and die much faster than other kinds of neurons because they are exposed to high Ca^2+^ loads during all life. In this scenario, PD is an inescapable result of extreme aging. PD may occur earlier in life in conditions that weaken the ability of mitochondria to cope with the high Ca^2+^ loads. This could happen because of mutations of key mitochondrial proteins or of the exposure to mitochondrial toxins [108]. Ca^2+^ oscillations may cause SNc damage also by another mechanism. L-type channels activation leads, indeed, to an increase in the synthesis of dopamine. The metabolism of this neurotransmitter with the generation of free radicals may further damage SNc neurons [109]. 

In neurodegenerative diseases Ca^2+^-dependent pacemaking may have roles independent from their involvement in cell death. For instance, T-type-dependent pacemaking in the subthalamic nucleus (STN) has a role in the genesis of the parkinsonian tremor [110]. STN is part of the indirect dopaminergic pathway. It has a role in the control of extrapyramidal movements. Therefore, it is often the target of the deep brain stimulation protocols used control PD symptoms [110]. Isolated STN neurons show repetitive single-spike firing. This spontaneous electrical activity depends on the interplay between voltage-gated Na^+^ channels and a Ca^2+^-dependent K^+^ conductance [110]. When they are hyperpolarized, STN neurons switch to burst firing [110]. Interestingly, the percentage of burst firing STN neurons significantly increases in experimental models of PD [111]. T-type Ca^2+^ channels maintain burst firing in STN neurons. Diverse T-type channel inhibitors including mibefradil, efonidipine or NNC 55-0396 block, indeed, STN burst firing in brain slices and in living rats undergoing single unit extracellular recordings [110]. In rats made parkinsonian by 6OH-DA these drugs normalize the percentage of burst-firing neurons [110]. Importantly, they also relieve the locomotor deficits observed in these animals [110]. These results suggest that the pharmacological blockade of T-type channels could decrease motor symptoms in PD. A drug acting on T-type channels, zonisamide (ZNS), was approved for PD even before the discovery of the role of these channels in STN. ZNS is an antiepileptic drug acting on multiple targets. It blocks voltage-gated Na^+^ channels, carbonic anydrase, MAO-B, and T-type channels [112]. In March 2009 ZNS was approved in Japan for PD. Drug approval was granted on the basis of the results of a multicenter, randomized, double-blind, parallel-treatment, placebo-controlled study showing the improvement in motor function upon ZNS add-on in patients not adequately responding to L-Dopa [113]. The effect of ZNS on PD motor symptoms was discovered by chance in a PD patient that was treated with this drug because of a concurrent seizure [114]. Since then, other clinical studies confirmed these initial findings. The mechanistic bases of the beneficial effects of ZNS in PD remain, however, obscure. The ability of the drug to reduce oxidative stress could have a role. ZNS, indeed, up- regulates manganese-superoxide dismutase (MnSOD) and inhibits monoamine oxidase B (MAO-B). These pharmacological effects contribute in protecting dopaminergic neurons from MTPT toxicity [115-118] and human neuroblastoma SHSY5Y cells from staurosporine-induced apoptosis [119]. Astrocytes could be responsible for ZNS neuroprotection. This drug increases, indeed, glutathione levels in these cells, but not in neurons [120]. Moreover, ZNS induces the expression of anti-oxidative and neurotrophic factors in astrocytes [121]. The new evidence that we reviewed above about the role of STN T-type channels in motor symptoms of PD strongly suggests that T-type channel blockade contributes to the favorable effect of the drug in PD. This hypothesis remains, however, to be proved. We would like to close this section with **essential tremor (ET), **another neurodegenerative disease whose symptoms depend on a T-type-dependent neuronal pacemaker. ET is a motor disorder characterized by tremor in the arms and hands during voluntary movements. In this disease, Lewy bodies occur in the brain stem, and cell loss or degenerative changes like torpedoes in cerebellar Purkinje cells [122, 123]. Therefore, ET is considered as a neurodegenerative disorder [122, 123]. The indole alkaloid harmaline produces a condition similar to ET in mice. In this experimental model, tremor is maintained by the synchronized oscillatory discharge of inferior olive (IO) neurons [124]. Pacemaking in these cells depends on Ca_V_3.1 T-type channels [124]. In agreement with these findings, harmaline treatment does not induce tremor in Ca_V_3.1 knock-out mice. This treatment induces 4-10-Hz oscillations in the IO in wild type but not in Ca_V_3.1 knock-out mice [124]. The local infusion of lentivirus harboring Ca_V_3.1-speciﬁc shRNA into the IO neurons also prevents harmaline-induced tremor in mice [124]. Intriguingly, the T-type channel blockers ethosuximide, zonisamide, KYS05064, and NNC 55-0396 and the neuroactive steroid (3β,5α,17β)-17-hydroxyestrane-3-carbonitrile (ECN), also ameliorate the neurological symptoms in experimental ET [125]. Several clinical studies evaluated ZNS in ET patients [126]. Authorities in the field feel, however, that the available evidence is inadequate to recommend ZNS in this disease [126].

### 2 VGCCs may be Toxic for Neurons Without Carrying Excess Ca^2+^

One of the most surprising findings emerging from recent work on VGCCs in neurodegeneration is that these ion channels may cause neuronal cell death even without transporting toxic Ca^2+^ loads. A decrease in the ability of VGCCs to carry Ca^2+^ ion may, indeed, kill neurons. Neurons may also die because VGCCs let toxic metal ion enter their cytoplasm. 

#### 2a Paradoxical Decrease of VGCC-Dependent Ca^2+^ Load in Neurodegenerative Diseases: the Case of Prion Disease 

We mentioned above that Ca^2+^ entry through L-type channels promotes neuron survival by activating neuroprotective Ca^2+^-dependent gene programs [15-18]. We also mentioned that a decrease in the activity of these channels could be responsible for delayed neuronal death in the ischemic penumbra [79]. Here we will focus on ***prion disease***, another condition where VGCC currents strongly decrease. The earliest evidence that prion protein could suppress VGCC was reported many years ago by Florio and coworkers [127]. They noted that, in GH_3_ pituitary cells, a synthetic peptide homologous to residues 106–126 of PrP (PrP_106–126_) suppressed L-type currents and the [Ca^2+^]_i_ response to high K^+^ solutions. These findings were later confirmed also in neurons [128]. Recently, Senatore *et al*. [129] suggested a molecular mechanism to explain prion effect on VGCCs. Briefly, they compared the [Ca^2+^]_i_ response to high K^+^ depolarization and the activity of VGCC currents in cerebellar granule cells from wild type and Tg(P14) mice [129]. These mice express a misfolded form of prion protein and develop a progressive fatal neurological disorders with ataxia, kyphosis, and foot clasp reﬂexes [129]. Both the [Ca^2+^]_i_ response to depolarization and VGCC current density were lower in transgenic than in control mice. These differences were already evident at the time of the first neurological symptoms, well before the histological evidence of neurodegeneration [129]. No change in VGCC gating properties was observed but the density of the channels in plasma membrane increased. The accessory α_2_δ subunits are crucial regulators of VGCC trafficking [130]. They are also part of the PrP interactome [131]. Therefore they are good candidates as effectors of prion modulation of VGCCs. Senatore *et al*. [129] showed that the α_2_δ-1 isoform physically interacts with mutant PrPs. Importantly, this causes VGCC retention in intracellular compartments and prevents their targeting to the plasma membrane [129]. The decrease in VGCC density at the plasma membrane causes a general decrease in synaptic transmission that could be responsible for the neurological symptoms observed in transgenic animals. The formal proof that the decrease in VGCC trafficking has also a role in causing neuronal cell death in prion disease is still missing. However, this seems a very likely possibility. Ca^2+^ influx through VGCCs is, indeed, crucial for neuronal survival [15-18] and neurons die of apoptotic cell death when exposed for many hours to CCBs [132]. Moreover, neuronal cell degeneration occurs in mutant mice showing spontaneous mutations or targeted deletions of the CACNA2D2 gene that encodes α_2_δ-2 subunits [133, 134]. Further studies are needed to establish whether drugs that increase VGCC currents could be effective in this devastating neurodegenerative disorder.

#### 2b Metal Ion Influx Through VGCCs 

**Fig. (3):**
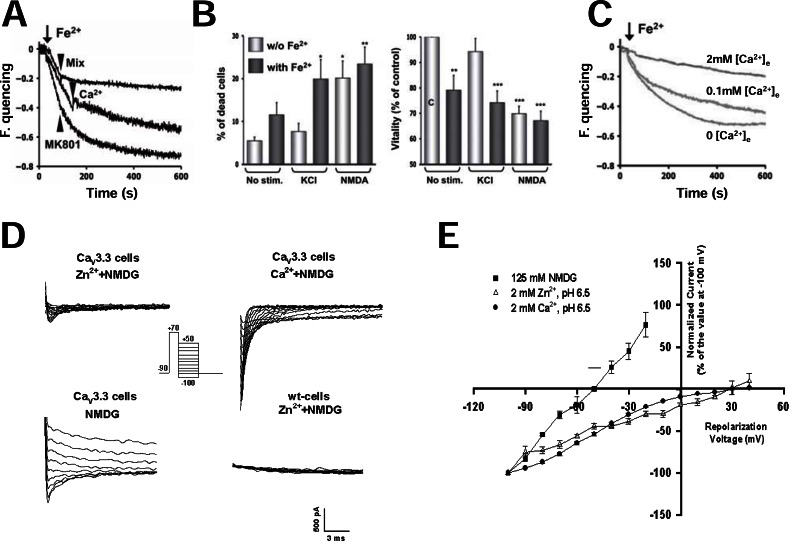
**VGCCs act as influx paths for metal ions.** *A*, *B* and *C*, Fe^2+^ ions enter the neuronal cytoplasm through VGCCs and cause cell death. *A*, the administration of 100µM Fe^2+^ to fura2-loaded hippocampal neurons incubated in a Ca^2+^ free extracellular solution causes fura2- fluorescence quencing. Fura2 quencing is unaffected by the NMDA blocker MK-801 (20µM), partially prevented by restoring physiological concentrations of extracellular Ca^2+^ (2mM) and virtually abolished by a mix of VGCC blockers including 10 µM nimodipine, 10 µM NNC 55-0396, and 1 µM ω-conotoxin MVIIC. *B*, fractional cell death increases and cell viability decreases in the presence of Fe^2+^ in the extracellular solution. Note that this detrimental effect of Fe^2+^ can be observed both in basal conditions and after cell depolarization with KCl. Interestingly, when hippocampal neurons are depolarized with KCl in the presence of extracellular Fe^2+^, fractional cell death increases up to values similar to those observed upon NMDA exposure. *C*, Fe^2+^-induced fura2 quencing is partially and dose-dependently reversed by increasing extracellular Ca^2+^ concentrations. (*A*, *B* and *C* reproduced with permission from Pelizzoni *et al.*, 2011 [150]). *D* and *E*, Zn^2+^ permeates through Ca_V_3.3 T-type channels. *D*, tail currents evoked by step repolarization of HEK-293 cells stably expressing Ca_V_3.3 channels to progressively more positive potentials after depolarizing the cell to +70 mV. The panel shows representative traces of the currents recorded in cells perfused with extracellular solutions containing, as indicated, 140 mM N-methyl-D-glucamine (NMDG) (to replace extracellular Na^+^) with no Zn^2+^ and no Ca^2+^ or plus either 2mM Zn^2+^ and no Ca^2+^ or 2mM Ca^2+^ and no Zn^2+^. For comparison, the traces recorded with Zn^2+^ in a control, untransfected HEK cell are also shown. All the recordings were performed at pH 6.5. *E*, reports the mean voltage to current plots of the experiments depicted in *D*. (*D and E*, unpublished data by Cataldi *et al.* presented in abstract form in ref. 162).

VGCCs have always been considered just Ca^2+^ influx systems. However, biophysical studies clearly show that other cations beside Ca^2+^ may permeate through these ion channels. The pore of L-type channels is large enough to let large organic cations permeate into the cell [135]. These cations may also permeate through T-type channels though their pore is smaller [83]. Importantly, also transition metals can flux trough VGCCs. This could be relevant in neurodegeneration because some of them including aluminum, manganese, iron and zinc have a role in the pathogenesis of major neurodegenerative disorders like AD, PD and multiple sclerosis (MS). These metals can cross the blood brain barrier through specific transporters and interact with neuronal VGCCs [136, 137]. Therefore, the hypothesis that VGCCs contribute to the toxicity of transition metals by carrying them into the neurons appears intriguing. Recent publications provide convincing experimental evidence that this could occur in the case of **iron**. This metal is essential for the normal functioning of the brain. It is necessary, indeed, for mitochondrial respiration and for the activity of many enzymes that synthesize or degrade the neurotransmitters [138]. The main among them are tyrosine hydroxylase, tryptophan hydroxylase, glutamate decarboxylase and glutamate transaminase, and the monoamine oxidases A and B [138]. However, when iron concentration increases too much, this metal becomes neurotoxic because it generates free radicals. The main mechanism by which this occurs is the conversion of Fe^2+^ into Fe^3+^ in the Fenton reaction. The free radicals so produced make the cells die by apoptosis [139]. Importantly, free radicals also cause the denaturation of specific proteins that precipitate and form protein aggregates. These are typical of many neurodegenerative diseases as in the case of PD in which Lewy bodies accumulate in neurons [140]. Protein aggregation and precipitation in intracellular compartments are an important cause of endoplasmic reticulum stress and, thus, of apoptotic cell death [140]. Recently, Dixon reported that iron causes a new form of non-apoptotic neuronal cell death known as ferroptosis in organotypic hippocampal slices challenged with glutamate [141]. Iron exerts its toxic effects after entering the neurons. In recent years, there was a considerable progress on how iron reaches these cells and enters their cytoplasm [142-144]. Briefly, iron, complexed with transferrin (Tf), crosses the blood brain barrier (BBB) upon binding to transferrin receptors (TfR) on endothelial cells. Once entered into the endothelial cell Fe^2+^ can be released into brain interstitial space in two different ways. First, it can exit the endothelial cells still bound to Tf. Second, it can leave their endosomes as Fe^2+ ^going through the divalent metal transporter-1 (DMT-1). Two different iron pools do exist in the interstitial space in the brain, iron bound to transferrin (Tf-I) and iron not bound to TF (NTBI). In NTBI, iron is bound to lactoferrin or melanotransferrin or, very loosely, to organic anions like lactate. Because extracellular iron concentrations in the brain exceeds the binding capacity of Tf [145], NTBI significantly contributes to brain iron homeostasis. Its role becomes crucial when iron load is increased or, as in PD, the concentration of Fe-binding proteins like Tf or neuromelanin is lower than normal. Tf-I enters the neurons by receptor-mediated endocytosis. The mechanism of NTBI uptake is, instead, controversial. Until recently, the prevalent view was that it requires the activation of DMT-1 [146]. This transporter is activated by NMDA receptors through a complex signaling cascade that involves the following steps. First, Ca^2+^ enters the cells through these receptors and causes nNOS activation. Then, a specific brain member of the ras family known as DEXras is S-nitrosylated. This leads to DMT-I activation by the AMP-kinase binding protein Pap7 [146]. Recent data strongly questioned the idea that DMT-1 is the only influx system for NTBI. Evidence has been reported, indeed, that VGCCs also represent a route of iron entry into the cytoplasm. This was demonstrated for the first time in the heart. In iron-overload cardiomyopathy, L-type Ca^2+^ channels are, indeed, a major patway for iron entry into cardiomyocytes [147, 148]. Gaasch and coworkers [149] demonstrated that a similar mechanism exists also in neuronal cells. They showed that membrane depolarization promotes the uptake of radioactive ^55^Fe into murine N2α neuroblastoma and in rat pheochromocytoma PC12 cells [149]. This effect was abrogated by the L-type Ca^2+^ channel blocker nimodipine [149]. This evidence suggested that L-type channels could be a influx path for iron in neurons. More recently, the group of F. Grohovaz in Milan, Italy confirmed and extended this conclusion [150]. They measured Fe^2+^ influx into the cytoplasm of hippocampal and cortical neurons with calcein fluorescence or fura-2 quenching [150]. Using this approach, they showed that basal and depolarization-induced iron influx is attenuated but not abolished by L-type channel blockade with nimodipine [150]. Instead, it was totally inhibited by an inhibitor cocktail contining nimodipine plus the T-type blocker NNC 55-0396 and ω-conotoxin MVIIC that blocks N-, P-, and Q-type channels [150] (Fig. **3A**). Therefore, not only L-type channels but also other subtypes of VGCCs may carry Fe^2+^ in neurons. Specifically, T-type channels could be involved. Intriguingly, these channels also contribute to cardiac iron overload in thalassemic mice [151]. Whatever the influx pathway involved, excess Fe^2+^ into the cytoplasm causes ROS generation, mitochondrial depolarization and cell death [150] (Fig. **3B**). This could explain why membrane depolarization with high K^+^, though usually ineffective in causing cell death in cultured neurons *in vitro*, becomes toxic in the presence of Fe^2+^ in the extracellular medium [150]. Therefore, the presence of high extracellular Fe^2+^ concentration should be added to the list of conditions that make potentially lethal VGCC activation in neurons. A key point that remains to be clarified concerns the role of extracellular Ca^2+^ in VGCC-dependent iron toxicity. Fe^2+^ ions are believed, indeed, to compete with Ca^2+^ for permeation. In keeping with this idea, the amount of Fe^2+^ ions entering the cell through these channels decreases as the extracellular Ca^2+^ concentration increases [149, 150] and it is maximal when extracellular Ca^2+^ concentration is well below its physiological value (Fig. **3C**). This raises the question of whether or not Fe^2+^ influx through VGCCs could be relevant in neurodegeneration *in vivo*. However, it should be pointed out that extracellular Ca^2+^ concentrations may decrease well below the “normal” values because of the sink activity of neurons both in conditions of intense synaptic stimulation as in epilepsy [152] or when damaged neurons become abnormally permeable to Ca^2+^ as in most of the neurodegenerative conditions [153]. Interestingly, evidence has been reported that L-type channel blockade with nifedipine could be effective in preventing iron accumulation in dopaminergic neurons also *in vivo* in rats treated with iron-dextran, an experimental model of iron overload [154]. 

Another neurotoxic metal that enters the neurons through VGCCs is Zn^2+^. In brain ischemia, it is coreleased with glutamate, enters the neurons and contributes to their death [88, 155, 156]. AMPA/kainate receptors are the main route of entry of Zn^2+^ in neurons [155]. However, VGCCs may also have a role [157]. Zn^2+^ influx in neurons is, indeed, promoted by membrane depolarization and blocked by CCBs and ω-conotoxin GVIA. In addition, in insect muscles fibers [158] and snail neurones [159], action potentials still occur when Zn^2+^ substitutes for Ca^2+^ in the extracellular solution. Also, patch clamp experiments showed Zn^2+^ currents in Helix neurones [160] and in mice cortical neurons [161]. This provided the direct demonstration that Zn^2+^ may permeate through HVA channels. Experiments performed with Zn^2+^-sensitive fluorimetric probes showed that VGCCs may carry this metal ion also in the presence of physiological Ca^2+^ concentrations [161]. L- and N-type channels are the VGCC subtypes involved in Zn^2+^ permeation. Depolarization-evoked Zn^2+^ influx in neurons was, indeed, blocked, by CCBs and ω-conotoxin GVIA. However, more recently, we showed that also recombinant Ca_V_3.3 channels may carry Zn^2+^ when Ca^2+^ is omitted from the extracellular solution [162] (Fig. **3D** and **F**). This suggests a role for T-type channels in Zn^2+^ permeation. Further studies are necessary to confirm this theory in neurons and prove its relevance in neurodegeneration.

### 3 VGCCs may Affect Neuronal Cell Survival Indirectly Through Actions Exerted on Nonneuronal Cells

The reason of the interest on VGCCs in neurodegeneration has been traditionally linked to the role that these channels may play directly in neurons. In the previous sections we examined the evidence indicating that VGCCs may act as a route of entry of toxic Ca^2+^ loads as it was supposed since the early days of the Ca^2+^ neurotoxicity hypothesis. In addition, we reviewed data showing that neuronal VGCCs may also take part to neurodegeneration without carrying excess Ca^2+^ either because they transport less Ca^2+^ than normal or because they carry metal ions inside the neuron. However, neuronal cell survival may also be indirectly affected by VGCCs through effects that these ion channels exert on non-neuronal cells. This represents an emerging and exciting field of investigation in neurodegeneration. Here we will go through some clear examples recently emerged in the literature that illustrate how VGCCs could be involved in the control of blood flow at the neurovascular unit and of immune responses by microglia. 

#### 3a Critical Role of VGCCs at the Neurovascular Unit

Cerebral blood flow matches the metabolic needs of neurons. This *neurovascular coupling* occurs thanks to the interaction of neurons, astrocytes, pericytes and vascular cells. These cell types form a highly integrated system known as the *neurovascular unit* [163, 164]. The *neurovascular unit* has also other physiological roles besides controlling brain perfusion. For instance, it controls BBB permeability, neurotransmitter availability in the extracellular space and brain inflammatory responses [161, 162]. Recent evidence suggests that pathological changes in the neurovascular unit contribute to the origin neurovascular and neurodegenerative disorders [165]. In this section we will report data on the role of VGCCs in the dysfunctions of the neurovascular unit in some of these conditions.

**Fig. (4):**
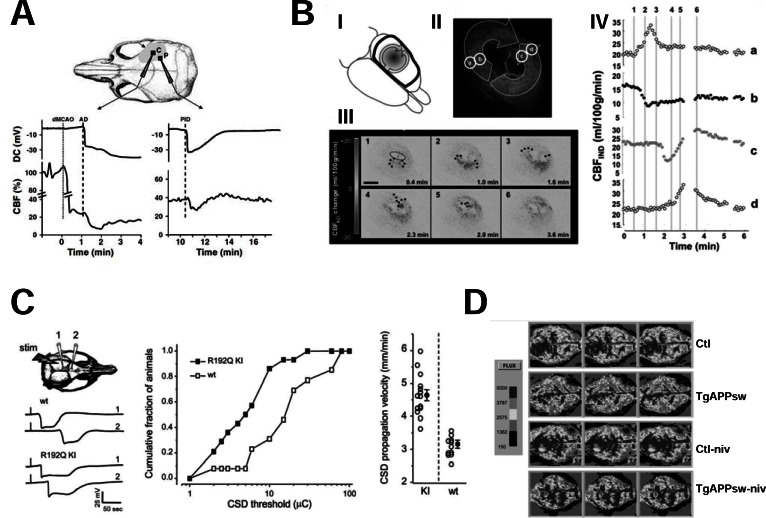
**Changes in CBF in neurodegenerative and neurovascular disorders and their modulation by VGCCs.** *A*, after MCAO, anoxic depolarization (AD) and postischemic depolarizations (PIDs) cause a marked drop in CBF. The two plots show the time course of DC currents and of CBF simultaneously recorded in the core (on the left) and in the penumbra (on the right) of the ischemic lesion caused by MCAO in a C57BL/6J mouse. The straight line in the left plot indicates the time of MCAO induction whereas the dotted lines correspond to the beginning of AD in the plot on the left, and to the beginning of PID in the plot on the right, respectively. The schematic draw on the top illustrates the position of the two extracellular recording electrodes. Note the sudden drop in CBF immediatly after AD in the core or PID, in the penumbra (reproduced with permission from Shin *et al.*, 2006 [166]). *B*, waves of vasoconstriction cycle around the ischemic core and contribute to enlarging the ischemic lesion. The arrows in the schematic draw in I represent the front of propagation of the changes in indicative CBF (CBF_IND_) occurring after MCAO in adult male Wistar rats. In II a laser speckle image from a representative animal is shown. Note that CBF_IND_ in the inner “core” region markedly decreases (black in II), that the region immediately surrounding the core (b and c in II) shows a hypoaemic response whereas a hyperemic response with an increase in CBF_IND_ increases occurs in the outer border of the lesion (a and d in II). These changes in CBF_IND_ propagate distally from the core in opposite directions thus originating two different fronts of propagation. As shown in III, these fronts of propagation (indicated by the black dots) cycle around the ischemic core and collide in a position opposite to their origin. The plot in IV reports the time course of CBF_IND_ in the different regions of interest a, b, c and d identified in II. Note that whereas in the outer regions (a and d) CBF_IND_ significantly increases (hyperaemic response), in the inner regions b and c a decrease in CBF_IND_ (hypoaemic response) is observed followed (as in c) or not (as in b) by a secondary hyperaemia (reproduced with permission from Nakamura *et al.*, 2010 [182]). C, Ca_V_2.1-encoded P/Q channels have a role in the propagation of CSDs. The plot on the left of the panel shows that CSDs last longer and propagate faster in mice harboring the R192Q migraine mutation in the Ca2.1 gene. The graph in the middle shows that the distribution of CSD threshold is right-shifted in R192Q Ca_V_2.1 transgenic mice as compared with controls whereas the scatter plot on the right reports the individual values of CSD propagation velocity in the different animals of the two groups (reproduced with permission from van den Maagdenberg *et al.*, 2004 [187]). *D*, nivaldipine restores normal CBF in a transgenic mouse model of Alzheimer’s disease. The panel shows two-dimensional maps of the regional blood flow measured with laser Doppler flowmetry in the cortex of 13 month old control mice (ctl), transgenic mice harboring the APP_K670N,M671L_ mutation (TgAPPsw), and control (ctl-niv) or TgAPPsw (TgAPPsw-niv) mice treated with nivaldipine (1 mg/kg of body weight daily for 15 days). Different CBF values correspond to different gray intensities as indicated in the gray scale reported on the left of the panel. Note that the very low CBF in TgAPPsw cortex is restored to values comparable to those of control mice upon treatment with nivaldipine. Conversely, this CCB does not modify CBF in control mice (reproduced with permission from Paris *et al.*, 2004 [223]).

Strong evidence suggests that a dysfunction of the neurovascular unit causes the secondary expansion of traumatic, hemorrhagic or ischemic **focal brain lesions**. In focal brain ischemia, the *ischemic core* immediately becomes hypoperfused and rapidly dies. On the contrary, neurons in the *ischemic penumbra* die hours later unless they are rescued by specific pharmacological interventions. Similarly, brain trauma and subarachnoid hemorrhage (SAH) cause a focal brain damage that gradually expands after the initial insult. In all these conditions, recurrent episodes of vasoconstriction occur for several days after the acute event [166, 167]. They are elicited by depolarization waves that originate in the core of the lesion and propagate into the neighboring healthy brain [168]. To be specific, immediately after a focal ischemic brain insult, a massive depolarization, the *anoxic depolarization*, arises in the ischemic core. The failure of the Na^+^/K^+^ ATPase causes its appearance. It induces, indeed, the accumulation of K^+^ ions in the extracellular space. This triggers the opening of multiple and still poorly defined cationic conductances [168]. In the core, anoxic depolarization causes cytotoxic edema. In addition, it forces the failing Na^+^/K^+^ ATPase to burn more ATP and further exhaust the cell of this nucleotide [168]. Anoxic depolarization does not remain secluded into the core [168]. Indeed, at the border between the ischemic core and the penumbra it elicits depolarization waves, known as *peri-infact depolarizations* (PIDs), that invade the penumbra at a speed of 2-5 mm/min [169]. PIDs propagate as a wave of negative deflection of extracellular direct current potential 10-20 mV wide [169]. Spreading depolarizations similar to PIDs propagate from the primary lesion into healthy brain after neurotrauma [170-173] or SAH [174]. The more generic term of *cortical spreading depolarizations* (CSD) is often used to refer to these depolarizing waves. Electrophysiologically, CSDs resembles the spreading depression that occurs during the aura of migraine attacks [175]. Indeed, they consist, of a large depolarization wave followed by a long lasting suppression of any spontaneous activity in the invaded network [168]. In stroke, CSDs recur for several days from the initial ischemic event and contribute to extend the ischemic damage [169]. This is suggested by the proportionality between the number and duration of PIDs and the growth of the ischemic lesion at the expenses of the penumbra [176-178]. Risher and coworkers used a two photon microscope to visualize the degenerative changes caused by PIDs in the cortical pyramidal neurons of a transgenic mice strain expressing GFP in these cells [179]. They observed, indeed, that rapid dendritic beading occurred after each PID episode [179]. Initially, neurons completely recovered at the end of each CSD [179]. However, after several CSDs dendritic damage became irreversible [179]. The mechanism by which CSDs cause brain damage is still a matter of debate. The massive neuronal depolarization caused by CSDs probably has a role. However, the prevalent view is that neuronal death depends on *spreading ischemia*. This term indicates waves of ischemia caused by the vasoconstriction elicited by PIDs. Shin and coworkers were the first to describe this process [166]. They simultaneously monitored cerebral blood flow with laser spekle flowmetry and extracellular potential with intracortical glass microelectrodes in mice undergoing middle cerebral artery occlusion (MCAO) [166]. Using this approach, they found that each PID causes a drop in brain perfusion. In addition, it increases by 140% the volume of the brain parenchyma in which blood flow is 20% or less than normal (Fig. **4A**). Strong *et al*. [167] reported similar findings in anestetized cats undergoing MCAO. CSDs extend focal brain damage also in humans [180]. This was demonstrated for the first time in patients with subarachnoid hemorrage (SAH). In this clinical condition the rupture of an aneurysm in the circle of Willis is followed by a vasospasm and ischemia downstream [180]. In his famous commentary on these findings in Nature Medicine, Costantino Iadecola [181] introduced the term *killer waves* to describes CSDs. This designation emphasizes the deadly potential of CSDs. Later studies demonstrated that CSDs extend brain damage also in stroke [182] and in neurotrauma [183]. Nakamura and coworkers showed that after stroke CSDs cycle around the ischemic core for several days and progressively enlarge the area of dead brain tissue [182] (Fig. **4B**). CSDs elicit different vasomotor responses in the healthy brain, as in migraineurs, and after focal brain damage. In the first case, they induce vasodilation, in the second, vasoconstriction. Therefore, the term of *inverse hemodynamic response* is often used to describe the vasomotor changes evoked by CSDs [168]. The reason of the different response of healthy and damaged brain to CSDs is unknown. It has been proposed, however, that it depends on the different levels of NO generated in these different conditions [184,185]. Because CSDs enlarge ischemic, traumatic or hemorrhagic focal brain lesions there is a major interest in identifying the factors responsible for their propagation or for the generation of the aberrant vasomotor responses that they elicit. Some data is emerging to suggest that VGCC could be involved. 

Specifically, recent evidence clearly point to P/Q VGCCs as important mediators of the propagation of CSDs into the penumbra. Considerable work performed on spreading depression in migraine provided clear evidence that its propagation involves P/Q channel activation. In particular, useful information came from the analysis of leaner and tottering mice. These two strains of mice carry mutations in the Ca_V_2.1 gene that hinder the activity of P/Q type channels. Both leaner and tottering mice have a high threshold for the induction of cortical spreading depression by KCl application on the pial surface or by electrical stimulation of the cortex [186]. On the contrary, mutant mice bearing migraine mutations that increase P/Q type channel activity have a high susceptibility to the same stimuli [187-189] (Fig. **4C**). Further arguments supporting the involvement of P/Q channels in spreading depression come from pharmacological experiments *in vitro*. Nonspecific VGCC blockers like Ni^2+^ and Cd^2+^ and selective P/Q type channel blocker ω-agatoxin-IVA blocked spreading depression elicited by electrical stimulation in a Na-acetate buffer in hyppocampal organotypic slices [190]. Instead, the L-type channel blocker nifedipine and the N-type channel blocker ω-conotoxin GVIA were ineffective [190]. P/Q channels also have a major role of ischemic CSDs. This was demonstrated by studies performed in transgenic mice with familial hemiplegic migraine type 1 (FHM1) mutations of the CACNA1A gene encoding for Ca_V_2.1. FHM is an autosomal dominant inherited form of migraine whose aura is characterized by episodes of hemiparesis [191]. As in other forms of migraine, FHM1 patients have a higher risk of stroke than age-matched controls and usually develop more serious forms of the disease [192, 193]. To explore the role the P/Q channels in ischemic CSDs, Eikermann-Haerter and coworkers [194] performed transient MCAO experiments in transgenic mice with the human R192Q or S218L FHM1 mutations of the CACNA1A gene [187, 188] and in their wild type littermates. Transgenic mice showed a higher frequency of PIDs recorded *in vivo* with intracortical microelectrodes than controls. These electrical events often occurred in clusters suggesting that, as in humans after stroke, their were cycling around the ischemic core [182]. Ischemic brain damage and mortality were also higher in mutant mice than in controls [194]. Importantly, the worse clinical course of stroke in transgenic mice respect to controls was related to a more rapid growth of hyperacute ischemic core [194]. These results suggest that stroke is more severe in mice with CACNA1A mutations because postischemic CSDs are enhanced. Another conclusion emerging from the cited study is that P/Q-type channels take part to the generation and/or propagation of postischemic CSDs. The mechanism involved remains, however, obscure. Given the role of this class of VGCCs in neurotransmitter release at the presynaptic terminal, the most likely explanation is that P/Q-type channel opening cause glutamate release in the ischemic brain. The activation of NMDA receptors is required, indeed, for PID propagation [195]. Consistent with this hypothesis, in FHM1 mice, excitatory neurotransmission is enhanced because of a higher probability of glutamate release at pyramidal cell synapses [196]. A potentiation in glutamate release could also explain why the CBF threshold for ischemic damage is lower in FHM1 mice than in controls [194]. Alernatively, P/Q type channels could affect post/ischemic CSDs by directly controlling the tone of brain microvessels. P/Q type channel are expressed, indeed, in brain vessels [197]. The role of P/Q type channels in the control of vascular tone was demonstrated in afferent glomerular arteries in the kidney but not yet in brain vessels [198, 199]. 

In **Subarachnoid hemorrage (SAH)**, the rupture of a subarachnoid aneurysm is followed by vasospasm in blood vessels of the circle of Willis. This causes brain ischemia in the region perfused by the constricted vessels. Vasoconstriction in SAH depends on Ca^2+^ influx into vascular smooth cells through VGCCs. L-type channels were originally identified as the class of VGCCs involved. Therefore, the use of CCBs was proposed to relieve vasospasm and prevent secondary cerebral ischemia in this disease. The current clinical evidence is solid enough to recommend the use of nimodipine in SAH [200]. However, the clinical response to this drug is often suboptimal. This led to the widespread perception that other mechanisms could be involved. Indeed, recent studies showed that other VGCC subtypes besides L-type channels are expressed in vascular smooth muscle cells [201, 202]. They contribute to a nimodipine-resistant component of vascular tone that becomes larger after SAH [202]. Our knowledge of the molecular choreography of ion channels in vascular smooth cells and, specifically, of VGCCs is still only partial. However, it is increasingly clear that a significant heterogeneity does exist among different vascular beds. Not only different ion channel subtypes do exist in different vascular districts but also different splicing variants of the same channels (e.g. L-type channels) could be expressed in different regions [102, 203]. L-type channels predominate in larger caliber proximal vessels [197, 202]. Instead, in smaller diameter resistance vessels, T-and R-type channels are also significantly expressed [197, 202, 204]. Intriguingly, SAH causes an increase in the expression in the basilar artery of the pore forming subunits of R-type channels, Ca_V_2.3, and of T-type channels, Ca_V_3.1 and Ca_V_3.3. On the contrary, the protein expression of the L-type channel subunits Ca_V_1.2 and Ca_V_1.3 decreases in the same condition [205]. The molecular mechanism responsible for the changes in T- and R-type channel expression in SAH is still unknown. However, the release of oxyhemoglobin from extravasated erythrocytes could have a role in this process. This protein promotes, indeed, the expression of R-type channels in isolated rat basilar arteries [206]. Recent evidence highlights the role in SAH of the parenchimal small resistance arteries that express R- and T-type channels. Specifically, cerebral ischemia and CSDs were observed in SAH patients also after the surgical placement of nicardipine prolonged-release implants in the subarachnoid space [207, 208]. In these subjects, there was no angiographic evidence of proximal vasospasm [207, 208]. 

A dysfunction of the neurovascular unit also occurs in neurodegenerative diseases not vascular in origin [209]. This happens, for instance, in **Alzheimer’s disease (AD)** that is now considered a progressive disorder of both neurons and brain vasculature [210, 211]. A severe decrease of regional brain perfusion occurs in transgenic mice models of the disease [212, 213] and in human patients [214]. This decrease in brain perfusion is a further argument to suggest that AD and cerebrovascular ischemic disorders are closely related (see section 1a). A weakening of cerebrovascular coupling (the ability to increase CBF in response to neuronal activity), cerebrovascular autoregulation (the ability to adjust vascular tone to compensate for changes in systemic blood pressure), and vasoreactivity to CO_2_ also occur in AD [215-217]. Vascular dysfunction in AD depends on amyloid deposition in blood vessel wall. This is part of cerebral amyloid angiopathy, a specific form of vascular pathology occurring in AD [218, 219]. Other features of this condition are small cortical infarcts and microhemorrages [218, 219]. βAP induces free radical generation, causes endothelial dysfunction and hinders the activity of endothelial NOS (eNOS) [220, 221]. This leads to an increase vascular tone [220,221]. Similar events also occur *in vivo*, in experimental animal models of AD [222]. The final vasoconstrictive response to βAPs requires the opening of L-type channels. Nivaldipine, indeed, prevents βAP_1-40_-induced vasoconstriction in isolated rat aortas [223]. It also normalizes CBF in the transgenic AD mouse model Tg APPsw [223] (Fig. **4D**). The beneficial effect of nivaldipine on regional blood flow has been demonstrated also in human AD patients [72]. Therefore, CCBs may be helpful in AD not only because of their direct neuroprotective effects but also because they prevent the effect of βAP on CBF. Nivaldipine has also other pharmacological properties that contribute to its neuroprotective effect. Indeed, it also decreases the synthesis of βAPs and increases their removal from the brain that is often low in AD patients [224, 225]. 

#### 3b N-Type Channels Control Chemokine Release from Microglia

In central nervous system, microglia can both cause tissue damage and promote healing [226-228]. In multiple sclerosis (MS), it has a role in the demyelination process [229]. It is well known that glial cells like astrocytes and oligodendrocytes express VGCCs [230-232]. On the contrary, their presence in microglia has been a matter of debate [233, 234]. Until recently, the only evidence of a role of VGCCs in microglial physiology was from pharmacological studies *in vitro*. Specifically, VGCCs take part in the [Ca^2+^]_i_ response to βAP_25-35_, PrP_106-126_, chemokines or HIV proteins in cultured microglia [235, 236]. Recently, evidence was reported of the relevance of these channels in microglial activation in living animals. Specifically, microglial N-type channels have been implicated in demyelination in experimental allergic encephalomyelitis (EAE), a model of MS. In animals with this disease, N-type channels accumulate in the plasma membrane of degenerating axons [237, 238]. Also, the N-type channel blocker ω-conotoxin GVIA protects Norway rats from EAE-induced optic neuritis [238]. Tokuhara and coworkers compared the clinical and pathological evolution of EAE in knockout mice for N-type channel pore forming subunit Ca_V_2.2 (α_1B_) and in their wild type littermates [239]. As expected, the neurological symptoms and the spinal cord lesions were less severe in knockout than in wild type mice [239]. Also perilesional inflammation was less severe in knockout mice. Fewer macrophages and microglial cells amassed, indeed, around the demyelinating lesions in knockout than in wild-type mice [239]. In these lesions, T-lymphocytes and microglial cells produce and release, respectively, monocyte chemotactic protein-1 (MCP-1) and macrophage inflammatory protein-1γ (MIP-1γ) [239]. These two chemokines further worsen tissue damage by recruiting inflammatory and immune cells to the demyelinating foci [239]. Importantly, less MCP-1 and MIP-1γ were released in knockout mice than in wild-type controls [239]. Ca_V_2.2 channels control MCP-1 release form microglia [239]. The N-type channel blocker ω-conotoxin GVIA suppresses, indeed, the release of this chemokine from microglial cells *in vitro*. Besides, MCP-1 synthesis and release is lower in cultured microglia from Ca_V_2.2 knockout than from control mice [239]. As a whole, these results suggest that N-type channels take part to microglia-dependent spinal cord damage in MS. Therefore, N-type channels should be added to the list of ion channels and transporters whose pharmacological modulation could be helpful in MS [240, 241].

## CONCLUSIONS

In conclusion, in neurodegenerative and neurovascular disorders, VGCCs influence cell survival in different ways (Table **2**). This may happen when their activity increases above average as in aging, in the presence of βAP or during chronic hypoxia. In addition, neurons showing Ca^2+^ dependent pacemaking are exposed to higher than average Ca^2+^ loads. Therefore, they are exquisitely vulnerable to neurotoxic insults. Also a decrease in VGCC activity below the normal may be detrimental for neurons. These ion channels, indeed, promote the influx of the Ca^2+^ ions that activate neuronal cell survival programs. VGCCs may kill neurons by letting toxic metal ions enter the cytoplasm. Finally, these channels may indirectly affect neuronal survival. In fact, they control vascular tone in the brain and the release of cytokines and chemochines by microglia. These data suggest that VGCCs could be a useful pharmacological target in neurodegenerative and neurovascular disorders. However, a major obstacle to developing effective VGCC-based therapies in humans is the lack of drugs that specifically act on dysfunctional brain VGCCs. This limit could be overcome by targeting the molecular mechanisms responsible for VGCC dysfunction in neurodegenerative diseases. This will represent a major challenge and, we hope, an active area of investigation in the years to come.

## Figures and Tables

**Table 1.** Molecular Diversity of VGCC

Current	Pore Forming Subunit	Localization	Specific Antagonists	Cellular Functions
**L**	Ca_V_1.1	Skeletal muscle transverse tubles	Dihydropyridines, phenylalkylamines, benzothiazepines	Excitation-contraction coupling
	Ca_V_1.2	Cardiac myocytes, endocrine cells, neuronal cell bodies and proximal dendrites	Dihydropyridines, phenylalkylamines, benzothiazepines	Excitation-contraction coupling, hormone release, regulation of transcription, synaptic integration
	Ca_V_1.3	endocrine cells, neuronal cell bodies and dendrites	Dihydropyridines, phenylalkylamines, benzothiazepines	Excitation-contraction coupling, hormone release, regulation of transcription, synaptic integration
	Ca_V_1.4	Retina	Not established	Neurotransmitter release from rods and bipolar cells
**P/Q**	Ca_V_2.1	Nerve terminals and dendrites	ω-agatoxin-IVA	Neurotransmitter release, dendritic Ca^2+^ transients
**N**	Ca_V_2.2	Nerve terminals and dendrites	ω-CTx-GVIA	Neurotransmitter release, dendritic Ca^2+^ transients
**R**	Ca_V_2.3	Neuronal cell bodies and dendrites	SNX-482	Repetitive firing
**T**	Ca_V_3.1	Neuronal cell bodies and dendrites	NNC 55-0396, R-efonidipine	Pacemaking, Repetitive firing
	Ca_V_3.2	Neuronal cell bodies and dendrites	NNC 55-0396, R-efonidipine	Pacemaking, Repetitive firing
	Ca_V_3.3	Neuronal cell bodies and dendrites	NNC 55-0396, R-efonidipine	Pacemaking, Repetitive firing

The table reports the current classification of the different VGCCs according to the type of Ca^2+^ current carried and of the gene encoding the pore forming subunit. For each class the main pharmacological properties and physiological roles are reported. Reproduced under permission with slight modifications from Catteral, W.A., Striessnig, J., Snutch, T.P., Perez-Reyes, E. International Union of Pharmacology. XL. Compendium of Voltage-Gated Ion Channels: calcium Channels. Pharmacol. Rev., 2003, 55(4), 579-581.

**Table 2.** Mechanisms of VGCC-Dependent Neurotoxicity

Cell Compartment Involved	Pathogenetic Mechanism	Diseases

**Neurons**		
with Ca^2+^ overload	Enhanced expression/activity of L-type Ca^2+^ channels	Ageing
		Chronic Hypoxia
		Alzheimer’s disease
without Ca^2+^overload	Ca^2+^-dependent pacemaker activity	Parkinson’s disease
		Essential tremor
	Pathological decrease of Ca^2+^influx through VGCC	Prion disease (?)
	Metal ion influx	Iron neurotoxicity
		Zinc neurotoxicity

** Neurovascular unit**	Enhanced generation/propagation of spreading depression	Ischemic Stroke, Neurotrauma, Subarachnoid hemorrage
	Enhanced vasomotor tone of small caliber brain blood vessels	Subarachnoid hemorrage

**Microglia**	Enhanced chemokine release	Multiple Sclerosis

## ABBREVIATIONS

AD: = Alzheimer disease
APP: = amyloid precursor protein
ASICs: = acid sensitive ion channels
β-AP: = β-amyloid peptide
BBB: = blood brain barrier
CBF: = cerebral blood flow
CCBs: = calcium channel blockers
cGMP: = cyclic GMP
CSD: = cortical spreading depolarizations
DMT-1: = divalent metal transporter-1
DIV: = days *in vitro*
EAE: = experimental autoimmune encephalomyelitis
eNOS: = endothelial nitric oxide synthase
ET: = essential tremor
FHM1: = familial hemiplegic migraine type 1
GSH: = glutathione
HIF-1: = hypoxia-inducible factor 1
HVA: = high voltage activated
IO: = inferior olive
LVA: = low voltage activated
MnSOD: = manganese-superoxide dismutase
MAO-B: = mono-aminooxidase B
MCAO: = middle cerebral artery occlusion
MCP-1: = monocyte chemotactic protein-1
MIP-1γ: = macrophage inflammatory protein-1γ
MPTP: = 1-methyl-4-phenyl-1,2,3,6-tetrahydropyridine
NCX: = sodium calcium exchanger
MS: = multiple sclerosis
NO: = nitric oxide
nNOS: = neuronal nitric oxid synthase
NTBI: = non/transferrin bound iron
PD: = Parkinson’s disease
PtdIns(3,5)P2: = phosphatidylinositol (3,5)-bisphosphate
PIDs: = peri-infact depolarizations
PS1: = presenilin-1
PKA: = protein kinase A
ROS: = radical oxygen substance
SNc: = substantia nigra pars compacta
STN: = subthalamic nucleus
6OH-DA: = 6-hydroxy-dopamine
Tet: = tetracycline
SAH: = subarachnoid hemmorage
Tf: = transferrin
Tf-I: = iron bound to transferring
TfR: = transferrin receptors
TRPM7: = transient receptor potential cation channel, subfamily M, member
VGCC: = voltage-gated calcium channels
ZNS: = zonisamide
